# HTLV-1 Rex: the courier of viral messages making use of the host vehicle

**DOI:** 10.3389/fmicb.2012.00330

**Published:** 2012-09-06

**Authors:** Kazumi Nakano, Toshiki Watanabe

**Affiliations:** Laboratory of Tumor Cell Biology, Department of Medical Genome Sciences, Graduate School of Frontier Sciences, The University of Tokyo Tokyo, Japan

**Keywords:** HTLV-1 Rex, retroviruses, post-translational regulator, CRM1, importinβ, B-23, HTLV-2, HIV-1 Rev

## Abstract

The human T-cell leukemia virus type 1 (HTLV-1) is a retrovirus causing an aggressive T-cell malignancy, adult T-cell leukemia (ATL). Although HTLV-1 has a compact RNA genome, it has evolved elaborate mechanisms to maximize its coding potential. The structural proteins Gag, Pro, and Pol are encoded in the unspliced form of viral mRNA, whereas the Env protein is encoded in singly spliced viral mRNA. Regulatory and accessory proteins, such as Tax, Rex, p30II, p12, and p13, are translated only from fully spliced mRNA. For effective viral replication, translation from all forms of HTLV-1 transcripts has to be achieved in concert, although unspliced mRNA are extremely unstable in mammalian cells. It has been well recognized that HTLV-1 Rex enhances the stability of unspliced and singly spliced HTLV-1 mRNA by promoting nuclear export and thereby removing them from the splicing site. Rex specifically binds to the highly structured Rex responsive element (RxRE) located at the 3′ end of all HTLV-1 mRNA. Rex then binds to the cellular nuclear exporter, CRM1, via its nuclear export signal domain and the Rex–viral transcript complex is selectively exported from the nucleus to the cytoplasm for effective translation of the viral proteins. Yet, the mechanisms by which Rex inhibits the cellular splicing machinery and utilizes the cellular pathways beneficial to viral survival in the host cell have not been fully explored. Furthermore, physiological impacts of Rex against homeostasis of the host cell via interactions with numerous cellular proteins have been largely left uninvestigated. In this review, we focus on the biological importance of HTLV-1 Rex in the HTLV-1 life cycle by following the historical path in the literature concerning this viral post-transcriptional regulator from its discovery to this day. In addition, for future studies, we discuss recently discovered aspects of HTLV-1 Rex as a post-transcriptional regulator and its use in host cellular pathways.

## INTRODUCTION

Human T-cell leukemia virus type 1 (HTLV-1) is widely accepted as the causative agent of adult T-cell leukemia (ATL) and was discovered almost a decade after the recognition of ATL as a disease (Takatsuki, 2005). By the early 1970s, many clinicians recognized the existence of a new type of human leukemia/lymphoma; however, an official description of ATL did not appear until 1977 in Kyoto, Japan. In 1979, HTLV-1 was confirmed in the United States (Gallo, 2005), and reported as the first human retrovirus (Poiesz et al., 1980, 1981). Soon after the discovery of HTLV-1, a retrovirus was also isolated from ATL patients in Japan and named adult T-cell leukemia virus (ATLV; Yoshida et al., 1982). It was then confirmed that ATLV and HTLV-1 were the same virus and the description was modified thereafter to indicate that ATL is caused by HTLV-1 (Popovic et al., 1982, 1983).

The genomic structure of the HTLV-1 provirus was thoroughly investigated and published by Seiki et al. (1983), which accelerated studies in biochemical and molecular aspects of HTLV-1 in the late 1980s and resulted in the first review on the molecular biology of HTLV-1 in 1995 (Franchini, 1995). Generally, RNA viruses have evolved elegant mechanisms to maximize coding potential and to precisely regulate the expression of encoded genes. Overlapping reading frames, internal ribosome entry sites, alternative splicing, sub-optimal Kozak sequences, and ribosomal frame shifting are among the varied mechanisms used to maximize genomic coding potential and regulate expression of specific viral genes (Balvay et al., 2007). HTLV-I has a compact genome RNA of 8685 nucleotides with two long terminal repeats (LTR) located at the 5′ and 3′ ends that function as the viral promoter. HTLV-1 encodes more than 10 open reading frames (ORFs) by employing several mechanisms to achieve appropriate and ordered expression of these genes, including alternative splicing and programmed ribosomal frame-shifting (PRF). In particular, *gag* and *pol* are separated by *pro*, which overlaps both the 3′ end of *gag* and 5′ end of *pol*. The protein precursors, Gag-Pro and Gag-Pro-Pol, share a common Gag initiator codon located at the 5′ end of *gag*, and expression is translationally regulated by an in-frame read-through and PRF. PRF is a mechanism frequently used by viruses to alter the translational reading frame by shifting the ribosome at a slippery site (Theis et al., 2008). The HTLV-1 RNA genome has a -1 PRF at nucleotide 1718 and another at nucleotide 2245. Moreover, HTLV-1 RNA genome contains two major splice sites. Unspliced HTLV-1 RNA yields Gag, Pro, and Pol proteins and the singly spliced RNA produces Env, whereas the functional proteins derived from the pX region can be translated only from doubly spliced mRNA.

The 3′ end of the HTLV-1 genome was named the pX region at the time the genomic structure of this virus was determined, since the function of this region was unclear. Deciphering the overlapped ORFs in the pX region allowed us to examine the encoded regulatory and accessory proteins of HTLV-1 in the pX region and newly discovered findings of wide-ranged functions of those viral proteins involved in the host cellular pathways have been quickly accumulated. Information concerning the function of HTLV-1 accessory proteins including Rex in the regulation of viral replication has been accumulated and updated during the last decade (Johnson et al., 2001; Franchini et al., 2003; Kashanchi and Brady, 2005; Taylor and Nicot, 2008; Kannian and Green, 2010). As a retrovirus, HTLV-1 is composed of only RNA genome that contains all the information necessary for self-replication; thus, the expression of viral genes entirely relies on the host transcriptional and translational machinery. Besides the structural proteins Gag, Pro, Pol, and Env, HTLV-1 encodes several unique regulatory and accessory proteins, such as Tax, Rex, P30II, p12, p13, and HTLV-1 basic leucine zipper factor protein (HBZ) coded in antisense ORF. Here we start this review of HTLV-1 Rex by introducing the functions of all viral accessory proteins before focusing on Rex, since these proteins function in concert to achieve successful infection and replication of HTLV-1 in the host cell. Thus, understanding the overall viral mechanism is necessary to understand the functional importance of Rex in the HTLV-1 life cycle.

## SCHEDULED AND CONCERT FUNCTIONS OF VIRAL PROTEINS FOR REGULATION OF VIRAL EXPRESSION

HTLV-1 has two major transcriptional regulators, Tax and Rex. Tax is a strong trans-activator of HTLV-1 LTR promoter, which enhances the expression of integrated HTLV-1 proviruses (i.e., viral replication) during the early phase of infection. Tax also has a significant influence on host signal transduction, gene expression, and cell cycle regulation by interacting with various cellular proteins and plays a major role in immortalization and leukemogenesis of the host T-cells (Matsuoka and Jeang, 2007; Boxus et al., 2008). On the other hand, it is also well recognized that Tax is expressed only during the early phase of infection and not expressed, at least not at a detectable level, thereafter. Consequently, it remains unclear how the “influence” of Tax is maintained for decades and triggers transformation of infected T-cells.

Rex is an mRNA binding protein, which specifically binds to the Rex responsive element (RxRE) and acts as a post-transcriptional regulator of HTLV-1 mRNA. Since RxRE locates to the U3 and R regions, all HTLV-1 transcripts (i.e., unspliced, singly spliced, and doubly spliced mRNA) have RxRE. The most important function of Rex is selectively binding to unspliced and partially spliced HTLV-1 mRNA in the nucleus and quickly exporting them to the cytoplasm, thereby preventing further splicing and enhancing effective translation of the structure proteins (Hidaka et al., 1988; Adachi et al., 1990, 1992; Hamaia et al., 1997).

A second HTLV-1 RNA binding protein, p30II, specifically binds to doubly spliced *tax/rex* mRNA and retains it in the nucleolus. Therefore, p30II reduces Tax and Rex expression levels (and thus, overall viral activity), which eventually leads the virus to enter the latent period (Nicot et al., 2004; Ghorbel et al., 2006; Sinha-Datta et al., 2007; Bai et al., 2010). Rex directly binds to p30II and rescues *tax/rex* mRNA retention by p30II to promote viral replication (Sinha-Datta et al., 2007); thus, switching between replication and latency is modulated by p30II and Rex interactions. In addition, p30II interacts with a number of cellular proteins and represses expression from HTLV-1 LTR by binding to p300, an important co-activator of LTR, probably by competing with Tax (Michael et al., 2006). This viral protein enhances the transforming activity of cMyc through interactions with a transforming co-activator, TIP60 (Awasthi et al., 2005). Recently, p30II was reported to enhance inappropriate DNA repair (Baydoun et al., 2011). The authors speculated that this new role of p30II may result in accumulation of DNA lesions during transformation of an infected cell. Anupam et al. (2011) also suggested an important role of p30II in enhancement of cellular survival under DNA damage through modulation of ataxia telangiectasia mutated (ATM) level, which is a key regulator of the cell cycle checkpoint initiated by a double-strand DNA break. The authors also demonstrated that REGγ, which stimulates the proteolytic activity of the 20S core proteasome independent of ubiquitination and ATP, unexpectedly enhanced p30II expression. Overall, p30II has multiple functions via interactions with both viral proteins/transcripts and cellular proteins and maintains a balance between viral latency and spread, as well as between cellular survival and transformation.

The small HTLV-1 accessory proteins, p12 and p13, are not essential for viral replication, but they play important roles in escaping from the host immune system and transformation of infected T-cells (Koralnik et al., 1993; Nicot et al., 2005). Finally, HBZ, a product of the antisense strand of HTLV-1 RNA genome, is known to promote viral replication and cellular proliferation (Matsuoka and Jeang, 2011) and induces T-cell lymphoma and chronic inflammation *in vivo* (Satou et al., 2011). The importance of this antisense-coded protein in the viral life cycle remains vague, although Arnold et al. (2006) showed that HBZ was dispensable for cellular immortalization *in vitro*, whereas it enhanced viral infectivity *in vivo* in a rabbit model. A new perspective of this antisense gene-coded product as a non-coding RNA was recently proposed, since HBZ has not been observed at detectable levels in HTLV-1 carriers and ATL patients, and a large portion of *hbz* mRNA was shown to accumulate in nucleus (Rende et al., 2011).

**FIGURE 1:**
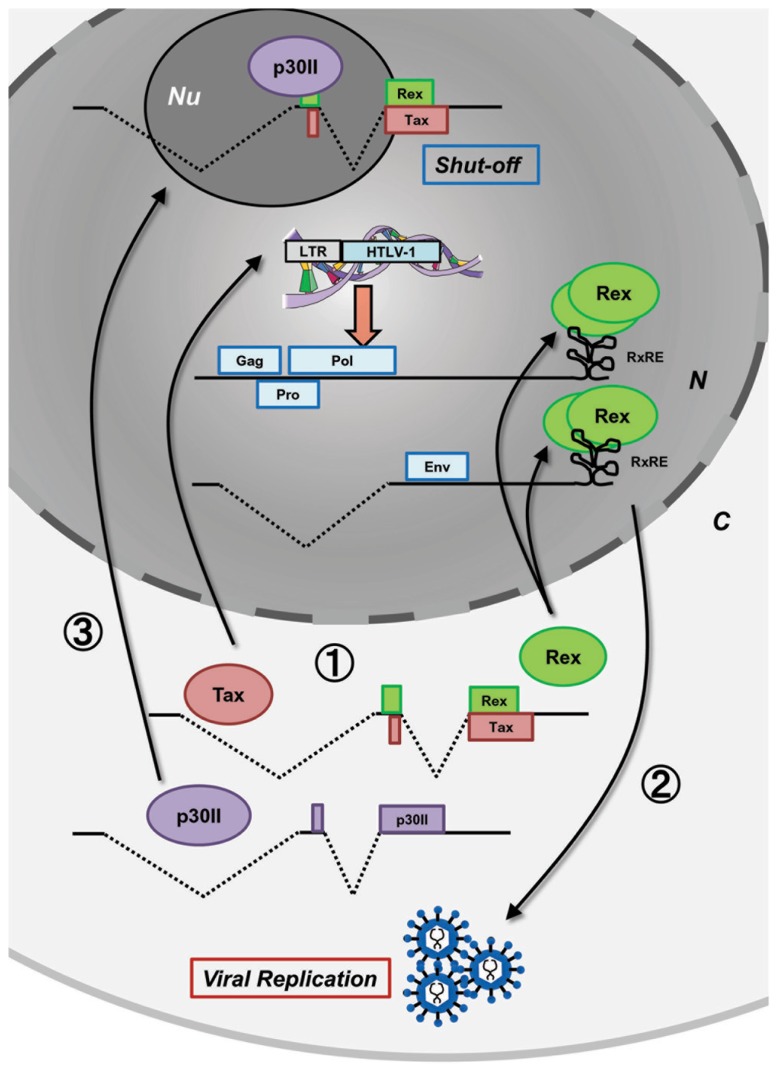
**Concerted functions of viral proteins for HTLV-1 expression.** Postinfection, the HTLV-1 provirus expresses viral proteins at appropriate times to control the early productive phase and the late shut-down phase leading to latency in the HTLV-1 life cycle. At the very beginning, without Rex, the viral transcripts are fully spliced and thus, Tax and Rex are selectively translated (stage 1). Tax transactivates HTLV-1 LTR promoter activity, whereas Rex inhibits splicing and actively exports the unspliced and singly spliced viral mRNA from the nucleus resulting in the expression of structural proteins and production of viral particles (stage 2). In the late phase, p30II from a minor, doubly spliced transcript binds to *tax/rex* mRNA and confines it to the nucleoli (stage 3) resulting in decreased Tax/Rex protein levels leading to latency. N, nucleus; C, cytoplasm; Nu, nucleolus.

After HTLV-1 entry and integration into the host human genome, proviral expression is initiated and the viral regulatory/accessory proteins function in concert with a precise schedule. Such well-organized regulation of HTLV-1 expression has been investigated by many researchers in the field of molecular and cellular virology and it was also recently confirmed by kinetic calculations (Corradin et al., 2010). **Figure 1** shows the time-course of HTLV-1 expression postinfection. Expression of the HTLV-1 provirus relies entirely on the host cell machinery and during the initial stage of infection, the viral mRNA is fully spliced to *tax/rex* mRNA. Since Tax has a stronger Kozak sequence than Rex, translation of Tax is initially superior to that of Rex (Green and Chen, 1990). Tax boosts transcription by LTRs and Rex gradually accumulates. Once a sufficient level of Rex is pooled in the host cell, Rex blocks splicing of viral mRNA and exports the unspliced and singly spliced viral mRNA to the cytoplasm for selective translation of Gag, Pro, Pol, and Env, resulting in active viral replication. Selective nuclear export of unspliced and partially spliced viral mRNA by Rex eventually reduces the export of fully spliced *tax/rex* mRNA, resulting in a decrease in Tax expression. Finally, p30II, with a strong nucleolar localization signal (NoLS), is expressed from the minor doubly spliced viral mRNA and retains *tax/rex* mRNA in the nucleoli, thus preventing their expression and avoiding immune evasion to initiate latency. The time course of HTLV-1 expression was thoroughly investigated by Li et al. (2009) in HTLV-1-expressing 293T cells. Such time-lagged operations of the positive (Tax and Rex) and negative (p30II) regulators of HTLV-1 promotes the early infectious phase followed by a rapid shut-down in the late infectious phase to escape from the host immune surveillance against pathogens (**Figure 1**).

During the course of viral expression, the small viral accessory proteins p13 and p12 also function to optimize the cellular environment for the viral spread and facilitate viral persistence in infected cells. p13, a short isoform corresponding to the C-terminal 87 aa of p30II is localized primarily in the mitochondrial inner membrane and increases mitochondrial permeability to K^+^ and activates the electron transport chain. This results in increased mitochondrial production of reactive oxygen species, which induces genetic instability and apoptosis (Silic-Benussi et al., 2010a,b; Biasiotto et al., 2010). p13 also localizes to the nucleus and is ubiquitinated by Tax for stabilization; thus, HTLV-1 balances viral expression and silencing through negative feedback (Andresen et al., 2011). The balance between T-cell activation and silencing is achieved by HTLV-1 p12 and p8, which are encoded in the singly spliced viral mRNA at minor splicing sites. p12, which mainly localizes to the endoplasmic reticulum (ER) and modulates T-cell activation and proliferation by interacting with the β and γ chains of the interleukin-2 receptor (IL-2R) and leading to activation of the Janus kinase/signal transducer and activator of transcription 5 (Jak/Stat5) signal transduction pathway to provide a mitogenic signal (Prooyen et al., 2010a,b). p12 also decreases surface expression of major histocompatibility complex I via proteasomal degradation, thus contributing to the rescue of HTLV-1-infected cells from being targeted by CTL. p12 also interacts with calreticulin and calnexin resulting in increased Ca^2^^+^ release from the ER and activation of the nuclear factor of activated T-cells (NFAT), a mitogenic pathway in T-cells. On the other hand, p8, which is cleaved from p12 in the ER, travels to the cell surface and induces T-cell anergy. p8 also increases cell-to-cell viral transmission through the formation of immunological synapses (Prooyen et al., 2010a,b).

HBZ was the first viral protein found to be encoded in the antisense ORF of HTLV-1. HBZ is known to interact with cAMP response element-binding protein 2 (CREB-2) and suppresses Tax-mediated viral transcription. HBZ also enhances viral replication (Matsuoka and Jeang, 2011). On the other hand, previous reports demonstrated that HBZ expression does not affect the ability of HTLV-1 to immortalize T-lymphocytes in culture (Arnold et al., 2006), and that *hbz* mRNA enhanced T cell proliferation in culture and transgenic mice (Satou et al., 2006). These reports proposed the possibility that HBZ proteins and *hbz* mRNA may have different functions. Choudhary and Ratner (2011) demonstrated that *hbz* mRNA destabilizes* p30ii* mRNA, thus increasing Tax expression. Rende et al. (2011) showed that *hbz* mRNA remains in the nucleus and speculated that *hbz* mRNA may have an important physiological role as a functional non-coding mRNA. Further investigations are necessary to clarify the involvement of HBZ and *hbz* mRNA in the HTLV-1 life cycle.

Overall, the interactions and positive and negative feedbacks among HTLV-1 Tax, Rex, p30II, and HBZ control the activation and inhibition of HTLV-1 expression, whereas p13, p12, and p8 organize a cellular environment suitable for viral retention.

## HTLV-1 Rex: THE CONDUCTOR OF VIRAL POST-TRANSCRIPTIONAL EXPRESSION

HTLV-1 Rex is a viral RNA binding protein of approximately 27 kDa and is essential for nuclear export of viral mRNA. Rex is also known to stabilize and export unspliced and singly spliced viral mRNA that code structural proteins; thus, Rex is considered essential for viral replication (Inoue et al., 1986, 1987; Hidaka et al., 1988; Gröne et al., 1996). It has been speculated that Rex interacts with the host splicing machinery in the nucleus to prevent splicing and stabilizes unspliced and partially spliced viral mRNA. However, the exact molecular mechanisms have not been fully elucidated to date.

As a viral post-transcriptional regulator, Rex binds to the RxRE of the viral transcript with high affinity. The RxRE sequence spans 255 nt from the U3 to R region of the 3′LTR and forms a stable secondary structure consisting of four stem loops (Ahmed et al., 1990). RxRE is not only a landmark for Rex binding, but it is also essential for optimal positioning of the polyA signal and polyA binding site in the HTLV-1 transcript, which are otherwise separated by the RxRE sequence (Ahmed et al., 1991). The *cis*-acting repressive sequence (CRS) is another regulatory sequence of HTLV-1 mRNA, located at both ends of HTLV-1 LTRs. Seiki et al. (1990) described the CRS in the U5 region for the first time and concluded that the CRS suppresses R activity, thereby enhancing RNA expression from the LTR. In agreement with their hypothesis, the authors demonstrated that the CRS in the U5 region significantly suppressed the expression of unspliced HTLV-1 mRNA only, but not spliced mRNA, since splicing within the R region removes the U5 element from the spliced mRNA. Interestingly, the function of Rex in protection of unspliced mRNA from splicing is CRS-independent. Thus, the CRS can be viewed as a post-transcriptional repressor, whereas Rex stabilizes unspliced viral RNA by directly interacting with the splicing machinery in addition to evacuating the unspliced viral mRNA to compartments not accessible to the splicing machinery. More recently, the other CRS in the 3′LTR region overlapping the RxRE sequence was identified by King et al. (1998). They examined the functions of 5′ and 3′CRSs separately and clarified that 5′CRS hampers nuclear export of only unspliced viral mRNA, whereas 3′CRS does so for all spliced and unspliced viral mRNA. This is rather reasonable, since 5′CRS remains only in unspliced mRNA, whereas 3′CRS is conserved in all forms of viral mRNA. They also found that deletion of both CRSs induced the constitutive nuclear export of reporter transcripts independent of Rex. Recently, Li et al. (2012) demonstrated that nuclear export of unspliced *gag/pol* mRNA and singly spliced *env* mRNA of HTLV-1 was Rex-dependent, whereas that of alternatively spliced mRNA was not. According to their conclusion, the unspliced and singly spliced HTLV-1 mRNA, containing RxRE/CRS and a functional splice donor site, are nuclear-exported in a Rex/RxRE-dependent manner, whereas the fully spliced mRNA is not, even though it contains a 3′RxRE/CRS. Their results are somewhat different from those of Bai et al. (2012), who demonstrated that *tax/rex* mRNA was also nuclear-exported in a Rex/RxRE/CRM1-dependent manner. All together, nuclear export of unspliced and spliced mRNA of HTLV-1 seems to be fine-tuned by nuclear retention activity of CRS and selective nuclear exporting activity of Rex.

Rex is a phosphoprotein; therefore, its activity is determined by the state of phosphorylation at the several serine/threonine residues (Kesic et al., 2009a). Adachi et al. (1990) demonstrated for the first time that Rex is activated by phosphorylation, since the treatment of an HTLV-1-infected cell line, HUT102, with a protein kinase C inhibitor, H-7 [1-(5-isoquinolinyl-sulfonyl)-2-methylpiperazine], resulted in decreased levels of unsliced viral mRNA and Gag-p19 protein. They also determined Rex phosphorylation sites at S70, S177, and Th174 (Adachi et al., 1992), although the kinase(s) responsible for Rex phosphorylation have not yet been identified. Recently, Kesic et al. (2009a) thoroughly examined Rex phosphorylation sites by conducting phosphoryl mapping and discovered five other phosphorylation sites at Thr-22, Ser-36, Thr-37, Ser-97, and Ser-106. On the other hand, they were unable to confirm the phosphorylation of Ser-177 as reported by Adachi et al. (1992) and concluded that Rex has seven phosphorylation sites in total. They also evaluated the importance of each phosphorylation site by a reporter assay using RxRE-dependent HIV-1 p24 Gag expression plasmids and concluded that phosphorylation of Ser-97 and Thr-174 most significantly influenced the expression level of the reporter plasmid, i.e., the RxRE-dependent nuclear export of reporter mRNA by Rex.

The HTLV-1 Rex, a protein of 27 kDa, contains several functional domains which play essential roles to induce the function of Rex as a nuclear–cytoplasmic mRNA transporter. The locations and physiological importance of each Rex domain are well described in several review articles (Younis and Green, 2005; Baydoun et al., 2008). A highly basic N-terminal RNA-binding domain located within aa 1–19 is essential for RxRE binding. This domain also serves as a nuclear localization signal (NLS), as well as a binding domain for p30II. The nuclear export signal (NES) spans from aa 66 to 118. Rex binds to Exportin-1 (CRM1), a cellular nuclear export protein through the NES; thus, this domain is essential for Rex function. The multimerization domains are located at the N- and C-terminal ends of NES (aa 57–66 and 106–124). The importance of NES and multimerization domains in Rex was well studied by Hakata et al. (1998), 2001. Based on a series of experiments investigating the interaction between CRM1 and Rex mutants in NES or in N′-multimerization domains, the authors found that NES is critical for interactions with CRM1. Thus, a multimer-deficient mutant Rex was translocated to the cytoplasm by CRM1; however, the multimer-deficient mutant Rex was not able to stabilize unspliced viral mRNA. Moreover, they revealed that rat CRM1 (rCRM1) was unable to support the function of Rex as an mRNA transporter because of its poor ability to induce multimerization of Rex, although rCRM1 can bind and export nuclear Rex proteins to the same extent as human CRM1. Accordingly, they concluded that the Rex protein needs to be both a multimerized and nuclear-exported to achieve its function, and that CRM1 was involved in multimerization and translocation of Rex. Recently, a stability domain was identified at the very end of the Rex C-terminus (aa 170–189; Kesic et al., 2009a,b; Xie et al., 2009). They showed that deletion of this segment resulted in a decreased half-life of Rex; however, the activity of Rex without the stability domain (SD), at least in translation from RxRE containing HIV-1 *p24 gag* mRNA, was not significantly influenced.

**FIGURE 2:**
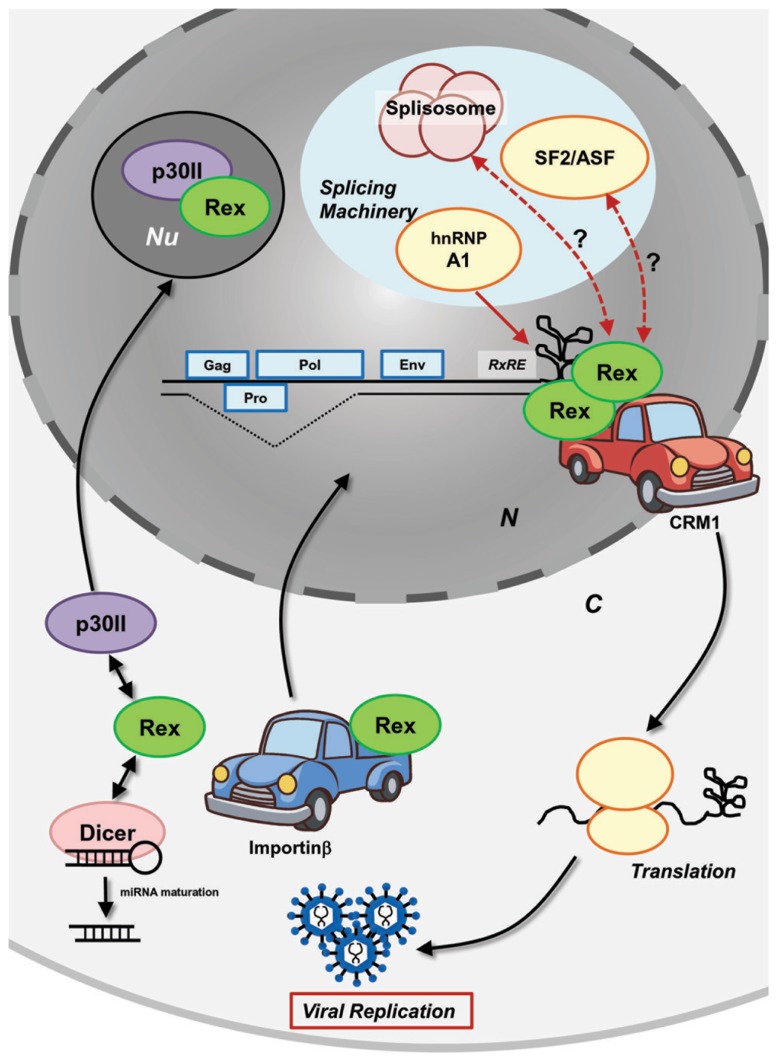
**Molecular mechanism of HTLV-1 Rex function.** HTLV-1 Rex specifically binds to the RxRE motif of HTLV-1 transcripts. Rex also interacts with the cellular nucleocytoplasmic shuttling protein, CRM1, through its NES. Consequently, the Rex–viral mRNA complex is exported from the nucleus by CRM1. In the cytoplasm, Rex subjects viral transcripts to the cellular translational machinery to enhance viral production. Released Rex binds to importinβ via its NLS and returns to the nucleus by the importin complex shuttling activity. P30II binds to Rex through its NLS and retains Rex in the nucleolus for suppression. Rex not only transports viral transcripts, but also inhibits splicing of viral mRNA that encode structural proteins. hnRNP A1, which governs the processing/splicing of pre-mRNA and transport of mature mRNA, was found to bind to RxRE in a competing manner against Rex. Another major splicing factor, SF2/ASF, was found to influence the processing of HTLV-1 mRNA (i.e., overexpression of SF2/ASF resulting in differential pX splice site utilization), although the direct physiological interaction to the viral proteins has not been examined. Recently, Rex was shown to directly interact with Dicer and inhibit its processing of shRNA to siRNA (Abe et al., 2010). Overall, interactions between Rex and other cellular mRNA processing proteins may lead to an unknown molecular mechanism of Rex in the inhibition of the splicing machinery. N, nucleus; C, cytoplasm; Nu, nucleolus.

To regulate viral expression through host machinery, Rex interacts with several host cellular proteins (**Figure 2**). To date, interaction of Rex with the following cellular proteins have been confirmed: CRM1 as already mentioned, the heterogeneous nuclear ribonucleoprotein A1 (hnRNP A1), the splicing factor SF2, importinβ, and nucleolar protein B-23. hnRNPs are heterogeneous nuclear RNA (hnRNA) binding proteins associated with pre-mRNA in the nucleus that influence the processing/splicing of pre-mRNA and the transport of mature mRNA. hnRNP A1 was shown to bind to the RxRE sequence of HTLV-1 viral mRNA in competition with Rex (Duc Dodon et al., 2002). Suppression of hnRNP A1 expression in HTLV-1-infected C91PL cells resulted in increased Rex-dependent nuclear export of unspliced and singly spliced mRNA, as well as in accumulation of unspliced mRNA (Kress et al., 2005). The authors confirmed that hnRNP A1 inhibits the function of Rex in a dose-dependent manner and proposed that hnRNP A1 may enhance the splicing processes of viral mRNA. Moreover, the authors found that the basal level of hnRNP A1 is lower in HTLV-1-producing cell lines (C91PL, MT2, and HUT102) when compared with non-HTLV-1-infected T-cell lines (CBL and Jurkat), indicating that HTLV-1 may induce the down-regulation of hnRNP A1, which is not conducive to viral replication. Another major splicing factor, SF2/ASF, also influences the processing of HTLV-1 mRNA, although direct physiological interactions with viral proteins have not been examined (Princler et al., 2003). SF2/ASF is considered to be involved in all splicing reactions in the cell and plays a critical role in splice site selection in a concentration-dependent manner. Indeed, overexpression of SF2/ASF resulted in differential pX splice site utilization, whereas hnRNP A1 caused HTLV-1 exon 2 skipping (Princler et al., 2003). HTLV-1-infected cells and ATL cells have different profiles of cellular transcripts, as they accumulate alternatively spliced transcripts compared to uninfected cells. Such observations may denote lesions in the splicing machinery in HTLV-1-infected cells.

Translocation of cellular proteins into the nucleus is due to interaction between *cis*-acting NLSs in the protein and nuclear transport receptor complex (the importin complex). Usually, importinα serves as a bridge between the NLS and the import receptor importinβ. It was demonstrated that the NLS of Rex directly bound to importinβ (Palmeri and Malim, 1999; **Figure 2**). The authors found that Rex was nuclear-imported by interactions with importinβ and independent of importinα. Nucleolar phosphoprotein B-23, also known as nucleophosmin (NPM), is a phosphoprotein mainly localized in nucleoli. Previously, it was determined that B-23 bound to the N′-terminal NLS/NoLS of Rex (Adachi et al., 1993). As described above, the Rex–viral mRNA complex is transported to the cytoplasm by CRM1. The authors speculated that B-23 may assist the return of Rex to the nuclei/nucleoli, which is necessary for further export of unspliced viral mRNA from the nucleus by Rex (Adachi et al., 1993). Recently, interactions between Rex and Dicer were reported by Abe et al. (2010). Their experiments demonstrated that Rex directly interacted with Dicer and inhibited its function in processing short hairpin RNA (shRNA) to small interfering RNA (siRNA).

## IMPACT OF Rex ON THE HOST CELLULAR HOMEOSTASIS

Viruses, including HTLV-1, utilize and direct host cellular mechanisms to facilitate viral replication through the whole life cycle. Such hijacking is achieved by direct interactions of viral and cellular proteins. The interactome and impacts of HTLV-1 Tax on the host cellular physiology have been well studied and described elsewhere, whereas those for Rex have not been thoroughly explored to date, even though numerous reports showed that Rex interacts with a wide variety of cellular proteins as mentioned above.

Rex up-regulates *il-2rα* mRNA expression, although the underlying mechanism has not been clarified. IL-2Rα overexpression in HTLV-1-infected and ATL cells influences the response efficiency to IL-2. Rex is capable of stabilizing *il-2rα* mRNA up to fivefold (Kanamori et al., 1990, 1994); thus, the overexpression of this gene in HTLV-1-infected and ATL cells can be explained, at least partly, by the function of Rex. White et al. (1991) found that the NoLS of Rex (aa 1–19) was critical for stabilization of *il-2rα* mRNA. The molecular mechanism of *il-2rα* mRNA stabilization by Rex still needs to be elucidated. If Rex stabilizes general mRNA metabolism of the cell, including that of *il-2rα* mRNA, it is highly possible that Rex influences the expression levels of other cellular transcripts.

Fyn is a proto-oncogene that belongs to the membrane-associated tyrosine kinase family and has been implicated in malignant pathological processes, especially in melanoma progression, neuroblastoma genesis, and carcinoma invasion. Compared to its implications in carcinogenesis, the physiological significance of Fyn in hematological malignancy has not been investigated. Fyn protein has two major isoforms, Fyn-B and Fyn-T, which are derived from exon 7A and 7B, respectively. Fyn-B is expressed in brain tissue, whereas Fyn-T is expressed exclusively in hematopoietic cells. Exon 7 of *fyn* encodes the linker region involved in intra-molecular interactions controlling Src tyrosine kinase regulation. Thus, the two isoforms have distinct functions in signal transduction and transforming capacity. Picard et al. (2004) reported that under pathological conditions, such as in acute lymphoblastic leukemia or chronic lymphocytic leukemia, expression of Fyn-B was significantly increased, as confirmed in cell lines and fresh patient cells. The author also mentioned that *fyn-b* mRNA levels are significantly increased in the HTLV-1-infected cell line, C91. Indeed, several years earlier, Weil et al. (1999) found for the first time that* fyn-b* mRNA is up-regulated in C91 cells and Rex is responsible for the down-regulation of alternative exon usage. Thus, abnormal exon selection of *fyn* mRNA is widely observed in various hematopoietic malignancies; however, the viral Rex protein may induce dysregulation in the host splicing machinery in HTLV-1-infected cells. The detailed molecular events explaining the implication of Rex in alternative splicing of Fyn and the physiological impacts of Fyn-B overexpression in T-cells have not been investigated. However, since Rex is an RNA binding protein, which has been implicated in the splicing machinery by several researchers, it is possible that Rex has the capacity to influence the splicing preference, resulting in an altered expression ratio of Fyn-B and Fyn-T in infected T-cells.

## SIMILARITIES AND DIFFERENCES BETWEEN HTLV-1 Rex AND HTLV-2 Rex

**FIGURE 3:**
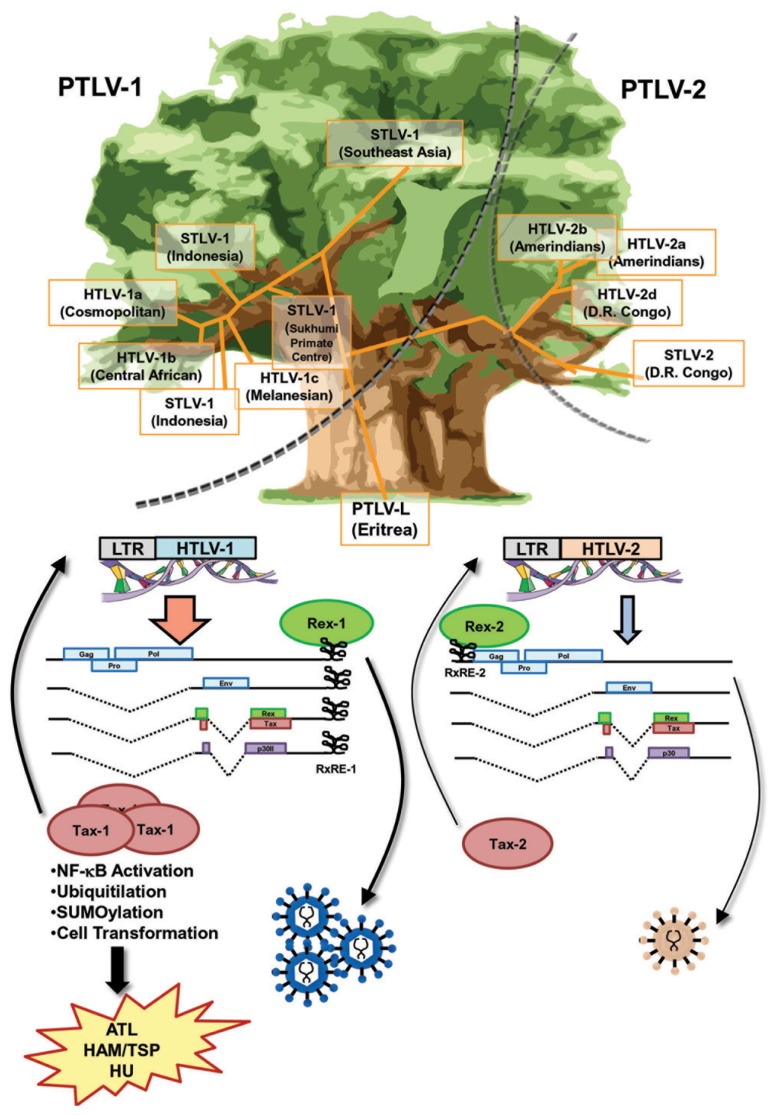
**Similarities and differences between Rex-1 and Rex-2.** The phylogenetic tree of HTLV, which is drawn based on the report by Vandamme et al. (1998), shows that the major branches of PTLV-1 and PTLV-2 separated at an early stage. Sub-branches of STVL-1 and HTLV-1 or STLV-2 and HTLV-2 were separated within each major branch thereafter. Yet, the genomic structures of HTLV-1 and HTLV-2 are very similar and both viruses encode Tax and Rex, the major transcriptional and post-transcriptional regulators, respectively. Both Tax-1 and Tax-2 have NLS, NES, and ATF/CREB binding domains, whereas only Tax-1 has a distinct NF-κB activating domain and p300 binding domain, as well as a number of PTM sites, for phosphorylation, ubiquitination, and SUMOylation, resulting in stronger transactivation and transforming activities than those of Tax-2. Both Rex-1 and Rex-2 are phosphoproteins sharing 60% similarity with overlapped major functional domains, such as NLS, NES, and SD at the 3′-terminus. The RxRE motif of HTLV-1 mRNA (RxRE-1) is located in the U3/R region and all HTLV-1 mRNA have intact RxRE-1s in the 3′UTRs. On the other hand, the RxRE of HTLV-2 (RxRE-2) is located at the R/U5 region and only unspliced mRNA maintains the intact RxRE-2. Thus, Rex-1 is capable of transporting all viral mRNA including *tax/rex* mRNA and enhancing the expression of Tax for further transactivation of LTR, whereas Rex-2 is not. Overall, Rex-1 may have a stronger impact on viral replication through the enhancement of Tax-1 expression compared with Rex-2. Different roles of Tax and Rex may be related to the differences in pathophysiologies of HTLV-1 and HTLV-2.

HTLV-1 and HTLV-2 belong to the same genus (Vandamme et al., 1998) and share a high homology in genomic structure (**Figure 3**). Both are able to infect human T-cells and induce immortality. In spite of a high similarity in the genome and life cycle, there is a significant difference in pathogenesis between retroviruses. The most outstanding difference is that HTLV-1 induces a severe hematopoietic malignancy (ATL), whereas HTLV-2 does not (**Figure 3**). It is unclear as to why there is such a significant difference in outcomes from similar genomic structures. Nevertheless, current knowledge indicates that the differences in properties and functions of accessory and regulatory proteins expressed from the pX region of the virus are critical for the distinct pathological differences between the HTLVs.

Both HTLV-1 and HTLV-2 encode Tax and Rex, the major transcriptional and post-transcriptional regulators. Tax-1 from HTLV-1 shows transforming ability, whereas Tax-2 from HTLV-2 does not. Thus, the different transforming activity of Tax determines the malignant pathology of this virus (Feuer and Green, 2005). Both Tax-1 and Tax-2 consist of NLS, NES, and ATF/CREB binding domains. On the other hand, only Tax-1 has a distinct NF-κB activating domain and p300 binding domain, as well as a number of post-translational modification (PTM) sites, such as phosphorylation, ubiquitination, and small ubiquitin-like modifier (SUMO)ylation (Rende et al., 2012). Generally, Tax-1 has stronger transactivation and transforming activities than Tax-2 (**Figure 3**).

Rex-1 encoded in HTLV-1 is a 27-kDa (189 aa) protein, whereas Rex-2 from HTLV-2 consists of 170 aa and its molecular weight ranges between 24 and 26 kDa depending on the phosphorylation-induced conformational changes (Kesic et al., 2009b; Xie et al., 2009). Rex-1 and Rex-2 share 60% similarity with overlapped major functional domains, such as RNA binding domain (RBD)/NLS at the N-terminus region, two multimerization domains, activity domain (AD)/NES, and SD at the 3′-terminus. In Rex-2, the inhibitory domain (ID) is overlapping with SD. Both Rex proteins are phosphoproteins and their activities are regulated by their phosphorylation status. Furthermore, Rex-1 and Rex-2 have isoforms derived from alternative splicing. p21Rex is the N′-truncated form of p27Rex, which lacks 78 aa of the N-terminus region, including RBD/NLS and the N′-multimerization domain (Kiyokawa et al., 1985). Alternative splicing inclusion of exon 2 yields p27Rex, whereas exon 2 skipping yields p21Rex (Orita et al., 1993). Since p21Rex does not have a NLS, it localizes to the cytoplasm. However, the functional importance of this isoform has not yet been elucidated. p21Rex transcripts are constitutively expressed in HTLV-1-infected cell lines and in primary peripheral blood mononuclear cells from HTLV-1 carriers and ATL patients (Berneman et al., 1992; Orita et al., 1992; Saiga et al., 1996). Thus, it is expected that p21Rex plays a role in the HTLV-1 life cycle, probably as a dominant negative form of p27Rex. Exon 2 skipping in HTLV-2 also yields N′-terminus-truncated forms of Rex-2 (tRex). Translation from the first AUG codon located within the x-III ORF results in two major protein isoforms of 22 and 20 kDa, as well as a minor protein isoform of 18-kDa depending on PTMs, whereas translation from the second AUG of the x-III ORF produces a 17-kDa protein (Rende et al., 2012). Ciminale et al. (1997) reported that tRex inhibited the function of the wild type Rex-2 by influencing the phosphorylation status and consequently, the subcellular localization of Rex-2.

A major difference between HTLV-1 and HTLV-2 regarding Rex function may be the position of RxRE in the viral transcripts (**Figure 3**). The RxRE motif of HTLV-1 mRNA (RxRE-1) is located in the U3/R region; consequently, all HTLV-1 mRNA have an intact RxRE-1 in the 3′UTR. On the other hand, the RxRE of HTLV-2 (RxRE-2) is located in the R/U5 region and only unspliced mRNA maintains an intact RxRE-2 (Rende et al., 2012). The principal function of Rex is selective nuclear export of unspliced or partially spliced viral mRNA. Recently, Bai et al. (2012) demonstrated that the nuclear export of the doubly spliced *tax/rex* mRNA of HTLV-1 was also enhanced by Rex-1 in a RxRE-1/CRM1-dependent manner. Considering the position of RxRE in the two HTLVs, Rex-1 may be capable of transporting all viral mRNA including *tax/rex* mRNA and enhancing Tax expression for further transactivation of LTR, whereas Rex-2 is not. Although Rex-1 and Rex-2 have similar capacities as RNA binding proteins, Rex-1 may have a stronger impact on viral replication through the enhancement of Tax expression (**Figure 3**).

The stability and efficiency of nuclear export of viral mRNA are determined by two *cis*-acting elements, RxRE and CRS, which function in a competing fashion. CRS is a nuclear retention signal that induces destabilization and inefficient nuclear-export of viral mRNA, although other proteins binding to CRS, either viral or cellular, have not yet been identified. The CRS is localized in the 5′LTR of HTLV-1 (Seiki et al., 1990) and HTLV-2 (Black et al., 1991). In both HTLVs, the 5′LTR CRS spans from the R region to the U5 region; thus, only unspliced viral mRNA contains intact CRS in either virus. HTLV-1 contains a second CRS at the 3′LTR overlap with RxRE-1 (King et al., 1998) resulting in all HTLV-1 mRNA containing intact RxRE-1 and CRS in the 3′LTR. The CRS overlaps with RxRE in both HTLV-1 and HTLV-2; therefore, it is possible that binding of Rex to RxRE might modulate the fate of viral mRNA (i.e., nuclear retention by CRS or nuclear export by Rex). Overall, it seems that Rex-1 might influence viral mRNA trafficking in a broader range compared with Rex-2 which targets only the unspliced *htlv-2* mRNA in terms of RxRE and CRS.

## SIMILARITIES AND DIFFERENCES BETWEEN HTLV-1 Rex AND HIV-1 Rev

**FIGURE 4:**
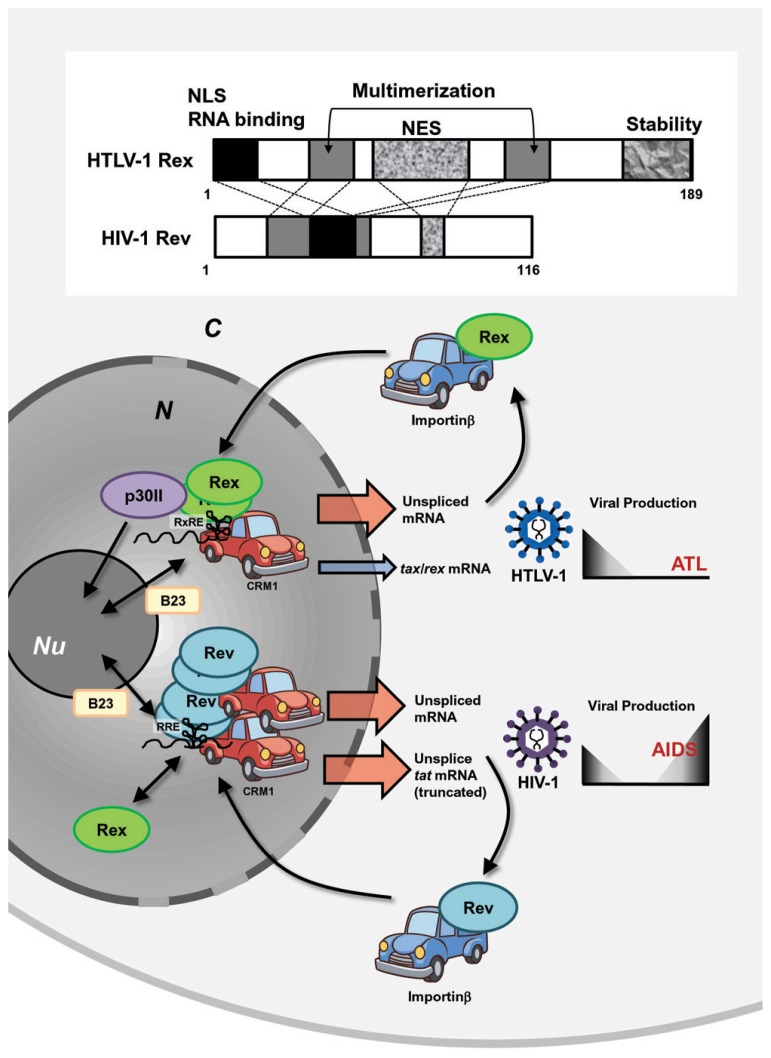
**Similarities and differences between HTLV-1 Rex and HIV-1 Rev.** HTLV-1 and HIV-1 are evolutionally distinct, but both belong to a family of complex retroviruses. Both viruses encode transactivators, HTLV-1 Tax and HIV-1 Tat, and post-transcriptional regulators, HTLV-1 Rex and HIV-1 Rev. Although Tax and Tat transactivate their respective LTRs, they act though different mechanisms and cannot be replaced with each other. On the other hand, even though the homology in the primary sequence of HTLV-1 Rex and HIV-1 Rev is low, they carry out similar functions through common domains, such as NES, NLS, and multimerization domains. They are RNA binding proteins and specifically bind to respective viral mRNA with high affinity through RxRE for Rex and RRE for Rev. They stabilize unspliced or partially spliced viral mRNA and actively transport them to the cytoplasm via CRM1 binding for selective translation of viral structural proteins and return to the nucleus by interactions with Importinβ. Rex can function through RRE, while Rev cannot bind to RxRE. HTLV-1 Tax is translated only from fully spliced viral mRNA; thus, stabilization and active nuclear transport of unspliced HTLV-1 mRNA by Rex reduces the relative expression rate of Tax. On the contrary, HIV-1 Rev does not suppress Tat activity, since it enhances truncated, yet active, Tat proteins. Thus, it is expected that HTLV-1 Rex favors reduction of viral production, whereas HIV-1 Rev may support active viral production. N, nucleus; C, cytoplasm; Nu, nucleolus.

HTLV-1 and HIV-1 are evolutionally distinct, but both belong to a family of complex retroviruses sharing tropism for human CD4^+^ T-cells. Although they have similar genetic structures and encode homologous viral proteins, the overall life cycle, controlled by viral accessory and regulatory proteins, are clearly different. This results in different disease associations [i.e., ATL by HTLV-1 and acquired immune deficiency syndrome (AIDS) by HIV-1]. Both viruses encode transactivators, HTLV-1 Tax and HIV-1 Tat, and post-transcriptional regulators, HTLV-1 Rex and HIV-1 Rev. Although Tax and Tat transactivate their respective LTRs, they act though different mechanisms and cannot be replaced by each other. On the other hand, even though the homology in the sequence of HTLV-1 Rex and HIV-1 Rev is low, they play similar functions through common cellular pathways (Baydoun et al., 2008; Suhasini and Reddy, 2009). A major similarity is that both Rex and Rev are RNA binding proteins and specifically bind to respective viral mRNA with high affinity through RxRE for Rex and the Rev responsive element (RRE) for Rev (**Figure 4**). Both Rex and Rev have arginine-rich sequences that are necessary for binding to their respective responsive elements. They stabilize unspliced or partially spliced viral mRNA and actively transport them to the cytoplasm for selective translation of viral structural proteins. The functional similarities and differences between HTLV-1 Rex and HIV-1 Rev were extensively investigated from late 1980s to the early 1990s.

Rimsky et al. (1988) first reported that the function of HIV-1 Rev could be replaced by that of HTLV-1 Rex. Rev induces translation of shorter forms of the Tat protein from the unspliced form of *tat* mRNA using a stop codon within the intron of *tat* mRNA, meaning that Rev suppresses splicing and stabilizes unspliced *tat* mRNA (Malim et al., 1988). Rimsky et al. (1988) also demonstrated that HTLV-1 Rex overexpression resulted in the stabilization of unspliced* tat* mRNA and enhanced translation of the truncated Tat protein. They also demonstrated that depressed viral production from HIV-1-δRev was rescued by co-transfection with HTLV-1 Rex-expressing plasmids. The authors emphasized the importance of the cellular post-transcriptional pathways for viral expression, which is shared by structurally distinct HIV-1 Rev and HTLV-1 Rex. Later, it was found that Rex functions through RRE (Hanly et al., 1989); however, Rex and Rev target different sequences within RRE (Solomin et al., 1990). Interestingly, although Rex can function through RRE, Rev cannot bind to RxRE (Hanly et al., 1989). Nevertheless, HTLV-1 Rex and HIV-1 Rev function through a similar mechanism for stabilization and active nuclear export of unspliced mRNA and the distinct genomic structures of these retroviruses furnish Rex and Rev with different expression levels of the transactivators Tax and Tat, respectively. Since Tax is translated only from fully spliced viral mRNA, stabilization and active nuclear transport of unspliced HTLV-1 mRNA by Rex eventually reduces the relative expression rate of Tax (Hidaka et al., 1988); thus, Rex might play an important role in the establishment of viral latency. On the other hand, HIV-1 Rev does not suppress Tat activity but enhances a truncated, yet active, Tat protein, as described above (Malim et al., 1988). Thus, the overall biological function of these viral post-transcriptional regulators in the viral life cycle may not be totally overlapped (**Figure 4**).

The arrangements of primary Rex and Rev structures are distinctive; however, both viral RNA binding proteins have NLSs and NESs and also use the same cellular nucleocytoplasmic shuttling machinery (Pollard and Malim, 1998; Kesic et al., 2009a; **Figure 4**). After translation, the NLSs of both Rex and Rev bind to importinβ and the complexes are then translocated to the nucleus (Palmeri and Malim, 1999; Truant and Cullen, 1999; Yoneda, 2000). Another key player of Rex/Rev nuclear import is B-23, a nucleolar phosphoprotein, and probably because of binding to B-23, these viral proteins localize strongly to the nucleoli. In the nucleolus, Rex and Rev bind to RxRE- and RRE-containing viral mRNA, respectively, and the viral RNA complex is exported to the cytoplasm for translation by CRM1 binding through NESs of Rex or Rev. Monomeric Rev has the highest affinity to RRE, but additional binding of up to 12 Rev molecules is required for effective nuclear export by CRM1 (Zapp et al., 1991; Zemmel et al., 1996). On the other hand, although monomeric Rex retains its ability to shuttle between the cytoplasm and nucleus, multimerization is essential for the function of Rex in stabilization and transport of viral unspliced RNA and CRM1 is involved in the multimerization process of Rex (Hakata et al., 1998, 2001; Baydoun et al., 2008). p30II, a negative post-transcriptional regulator of HTLV-1 (Nicot et al., 2004), has multiple NoLSs, and retains *tax/rex* mRNA as well as Rex proteins in the nucleoli. Therefore, p30II is considered to suppress HTLV-1 expression (Ghorbel et al., 2006; Sinha-Datta et al., 2007). There is no counterpart of p30II in HIV-1. Thus, it can be speculated that Rev alone has a NoLS strong enough to retain itself in nucleoli and multimerization is necessary for interacting with multiple CRM1s to be exported from the nucleolus. On the other hand, Rex may have less powerful NoLSs and without p30II, monomeric Rex can be exported by CRM1, although multimerization is necessary for this protein to interact with RxRE-containing viral RNA (**Figure 4**).

Involvement of both Rex and Rev in the cellular splicing machinery is expected, since both protect unspliced viral RNA. HIV-1 Rev strongly interacts with the splicing co-factor p32 (Tange et al., 1996). The p32 protein is one of three polypeptides composing active SF2/ASF in HeLa cells, which are involved in many splicing events and are required for splice site selection in a concentration-dependent manner (Krainer et al., 1991). Later, SF2/ASF was also shown to bind to RRE in a Rev-dependent manner (Powell et al., 1997). Therefore, p32 may function as a bridge between Rev and SF2/ASF to recruit an optimal amount of SF2/ASF to RRE in order to inhibit splicing of HIV-1 mRNA, although the molecular mechanism of Rev in inhibition of splicing has not been fully clarified.

HTLV-1 Rex is also known to inhibit the early phase of splicing (Younis and Green, 2005) through interactions with SF2/ASF, although the pathways have not been extensively examined compared with HIV-1 Rev. hnRNA binding proteins (hnRNPs) associated with pre-mRNA in the nucleus influence pre-mRNA processing/splicing and transport of mature mRNA. hnRNP A1 was demonstrated to bind to RxRE in a competing manner to Rex (Duc Dodon et al., 2002) and inhibit the function of Rex (Kress et al., 2005). The authors found that the basal level of hnRNP A1 was lower in HTLV-1-producing cell lines (C91PL, MT2, and HUT102) compared with non-HTLV-1-infected T-cell lines (CBL and Jurkat), proposing that HTLV-1 may have evolved a mechanism to down-regulate hnRNP A1 because it is not beneficial to viral replication. Several reports indicated that Rex was involved in post-transcriptional regulation of the host genome. For example, Rex stabilizes *il-2ra* mRNA, with its NLS playing an important role (Kanamori et al., 1990; White et al., 1991). Further, Rex enhances the alternative usage of exon 7 in *fyn* mRNA to yield the brain-type Fyn-B, instead of T-cell-type Fyn-T (Weil et al., 1999). The underlying mechanism by which Rex influences cellular post-transcriptional regulation has not yet been fully clarified. It is possible that Rex interacts with cellular splicing factors to enhance viral replication, which may cause incidental alterations in host transcriptional homeostasis.

Although HTLV-1 Rex and HIV-1 Rev are structurally distinct, they have evolved a similar function, i.e., inhibition of splicing and stabilization and nuclear-export of unspliced viral mRNA through interactions with common cellular factors. On the other hand, the difference between these two post-transcriptional regulators might be reflected in the different pathophysiological characteristics of HTLV-1 and HIV-1.

## NEW TOPICS IN HTLV-1 Rex MOLECULAR BIOLOGY FROM RECENT STUDIES

Cellular physiological pathways are achieved by functional combinations of cellular proteins. It has been clarified that such protein–protein interactions are achieved through short linear motifs (SLiMs) consisting of 3–13 aa, rather than large structural domains of each protein (Davey et al., 2012). Interestingly, SLiMs were first identified in viruses and it was discovered later that the viruses actually mimic the functional motifs of cellular proteins to hijack the cellular pathways (Kadaveru et al., 2009; Davey et al., 2011). SLiMs participate in all aspects of cellular biology, such as protein–protein binding (SH3 domain interactions), targeting (NLS and NES), PTMs (phosphorylation, SUMOylation, and ubiquitination), and cleavage, which also overlap with the viral life cycle from entry to budding in the host cells. However, viral mimicry of host SLiMs has not been fully investigated. Davey et al. (2011) reviewed 52 viral mimicry instances among approximately 150 reported eukaryotic motifs in human papillomavirus (HPV), Epstein–Barr virus (EBV), human T-cell lymphotropic virus (HTLV), adenovirus, human immunodeficiency virus (HIV), and influenza virus. Nevertheless, the authors were expecting more extended mimicry by viruses. The well-known viral mimicry of HTLV-1 Rex involves NLS and NES in cellular nucleocytoplasmic shuttling. Since this viral post-transcriptional regulator extensively functions by means of host cellular pathways in various steps of the HTLV-1 life cycle, Rex may have other mimicry motifs that have not yet been discovered.

**FIGURE 5:**
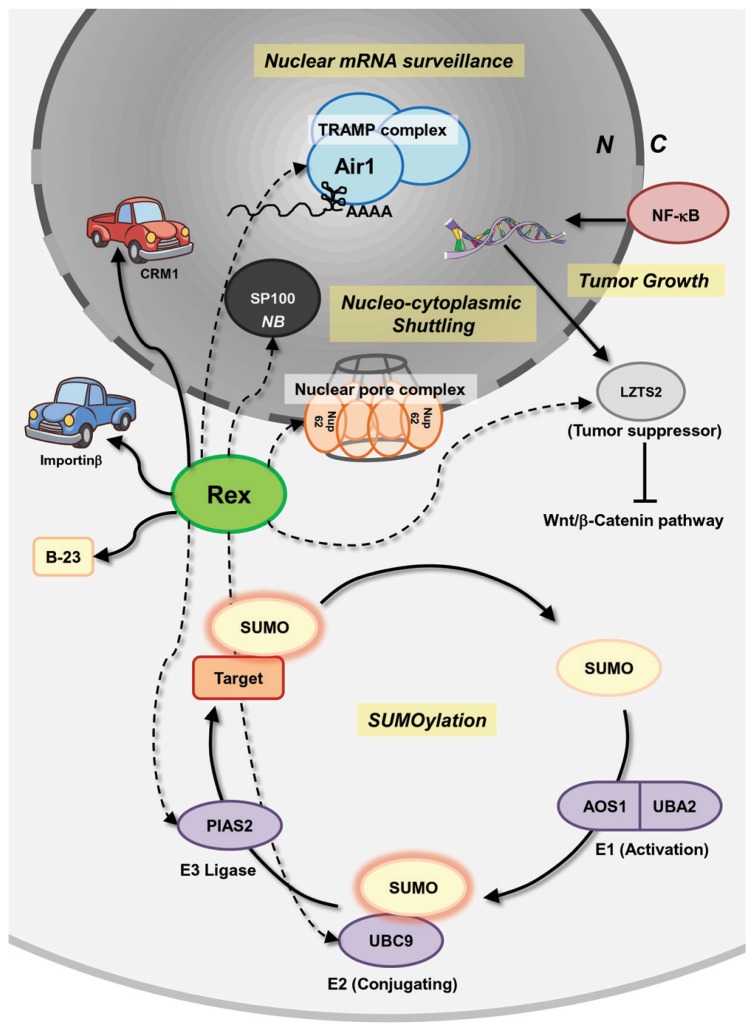
**Interactions of Rex with host pathways, uncovered and covered.** Besides the known interactions between Rex and CRM-1, importinβ, and B-23, a number of potential interactions between Rex and cellular proteins, based on the high-throughput yeast two-hybrid system, were reported by Simonis et al. (2012). Rex is suspected of interacting with a series of proteins that play crucial roles in mRNA surveillance, nucleocytoplasmic shuttling, tumor growth regulation, and SUMOylation. Air1 (ZCCHC7) is a component of the TRAMP complex, which is involved in nuclear mRNA surveillance. NUP62 is one of three nucleoporins (NUP54, 58, and 62) composing the nuclear pore complex and is essential for nuclear transport. LZTS2 is a tumor suppressor and its expression is transcriptionally regulated by NF-κB. LZTS2 expression levels affect cell proliferation and tumor growth through the Wnt/β-catenin pathway in various cancer cell lines. An E2 SUMO ligase, UBC9, and an E3 SUMO ligase, PIAS2, are also expected to interact with Rex. Rex is also expected to interact with SP100, a major component of the nuclear body, which has a transactivating function and is induced in stimulated and malignant cells. Since Rex impacts the host cell through an unknown mechanism, such as increasing *il-2r*α mRNA level and *fyn-b* mRNA expression level by enhancing unusual exon 7 usage, unknown interactions between Rex and cellular proteins may be related to Rex functions. Solid lines indicate reported interactions. Dashed lines indicate potential interactions. N, nucleus; C, cytoplasm; NB, nuclear body.

Recently, comprehensive interactomes, based on the high-throughput yeast two-hybrid system (Rual et al., 2005; Venkatesan et al., 2009), between HTLV-1/HTLV-2 viral proteins and human proteins were reported by Simonis et al. (2012). The authors discovered (including confirmation of previous reports) 87 and 79 interactions between HTLV-1- and HTLV-2-encoded proteins, respectively, and 122 human proteins participated in Ub-proteasome pathways, apoptosis, oncogenesis, and Notch signaling. For HTLV-1 Rex, 18 novel interactions were identified, including an interaction with Dic2 (Rho-Gap protein) and BHLHB2 (a transcription repressor) having an anti-apoptotic function. Recently, it was demonstrated that BHLHB2 mediated HIF-1α-induced microphthalmia-associated transcription factor (MITF) suppression, which causes increased metastasis in melanoma cells (Cheli et al., 2011). In addition, Rex is suspected of interacting with a series of proteins that play crucial roles in mRNA surveillance, nucleocytoplasmic shuttling, tumor growth regulation, and SUMOylation (**Figure 5**). The cellular proteins listed below potentially interact with Rex. Air1 (ZCCHC7) is a component of the Trf4/Air2/Mtr4 polyadenylation (TRAMP) complex, which is involved in nuclear mRNA surveillance (Fasken et al., 2011). NUP62 is one of three nucleoporins (NUP54, 58, and 62) composing of the nuclear pore complexes that are essential for nuclear transport (Solmaz et al., 2011). The interaction between viral proteins and NUP62 has been reported in HIV-1, herpes simplex virus, and EBV. In HIV-1, it is speculated that Rev reorganizes the architecture of nuclear pore complexes, including NUP62, for efficient viral RNA transport (Monette et al., 2011). In addition, HIV-1 integrase interacts with NUP62 on chromatin for integration of the viral genome (Ao et al., 2012). The HCV post-transcriptional regulator ICP27 was demonstrated to directly bind NUP62 to inhibit cellular trafficking and increase viral mRNA transport (Malik et al., 2012). Finally, EBV BGLA4, a viral serine/threonine kinase, was shown to interact with NUP62 and NUP153 and translocate itself to the nucleus even though this protein does not have any clear NLSs (Chang et al., 2012). LZTS2, a tumor suppressor, which is transcriptionally regulated by NF-κB, and the modulation of LZTS2 expression affects cell proliferation and tumor growth through the Wnt/β-catenin pathway in various cancer cell lines (Kim et al., 2011). An E2 SUMO ligase, UBC9, and an E3 SUMO ligase, PIAS2, are also expected to interact with Rex. SUMOylation is a major PTMs (Seeler and Dejean, 2001; Gareau and Lima, 2010), which modulates the function of a large number of proteins, but its dysfunction is closely related to pathogenesis (Wimmer et al., 2012). Rex also reportedly interacts with SP100, a major component of a nuclear body (NB), which has a transactivating function and is induced in stimulated and malignant cells. The function of SP100 in modification of molecular dynamics of a NB is regulated by SUMOylation (Riley et al., 2005; Bossis and Melchior, 2006 As shown in **Figure 5**, there is a wide variety of cellular proteins that potentially interact with Rex. Taken as a whole, HTLV-1 Rex has a great potential to be involved in or even direct unknown cellular pathways.

## CONCLUSION

HTLV-1 Rex is a major post-transcriptional regulator of viral expression, which is responsible for active viral replication in the early phase of infection and for reduction of viral activity to establish latency in the late phase of infection. The molecular biology of Rex was extensively investigated for a decade from the 1980s to the early 1990s; however, once the molecular mechanisms of nuclear export of unspliced viral mRNA by Rex was clarified, the major interest was shifted to the function of Tax to understand HTLV-1 virology and pathology. Nevertheless, our understanding of various aspects of HTLV-1 Rex inside and outside of the viral life cycle is incomplete. For example, it is unclear how Rex inhibits splicing of viral mRNA (and probably the host mRNA), and the extent of the influence of Rex by making use of the cellular pathways for viral benefits. We still do not know the underlying mechanism by which Rex increases *il-2rα* mRNA or the impacts on the host cell caused by unusual exon-usage for production of Fyn-B. Several reports already proposed the possibility of unknown biology of HTLV-1 Rex. Detailed and extended investigations based on uncovered facts and recent knowledge may open new pathways to discover hidden aspects of HTLV-1 Rex.

## Conflict of Interest Statement

The authors declare that the research was conducted in the absence of any commercial or financial relationships that could be construed as a potential conflict of interest.
